# Embryonic Cerebrospinal Fluid Nanovesicles Carry Evolutionarily Conserved Molecules and Promote Neural Stem Cell Amplification

**DOI:** 10.1371/journal.pone.0088810

**Published:** 2014-02-12

**Authors:** David M. Feliciano, Shiliang Zhang, Carole M. Nasrallah, Steven N. Lisgo, Angélique Bordey

**Affiliations:** 1 Departments of Neurosurgery, and Cellular & Molecular Physiology, Yale University School of Medicine, New Haven, Connecticut, United States of America; 2 Institute of Genetic Medicine, Newcastle University, International Centre for Life, Newcastle upon Tyne, United Kingdom; Université de Technologie de Compiègne, France

## Abstract

During brain development, neural stem cells (NSCs) receive on-or-off signals important for regulating their amplification and reaching adequate neuron density. However, how a coordinated regulation of intracellular pathways and genetic programs is achieved has remained elusive. Here, we found that the embryonic (e) CSF contains 10^12^ nanoparticles/ml (77 nm diameter), some of which were identified as exosome nanovesicles that contain evolutionarily conserved molecules important for coordinating intracellular pathways. eCSF nanovesicles collected from rodent and human embryos encapsulate protein and microRNA components of the insulin-like growth factor (IGF) signaling pathway. Supplementation of eCSF nanovesicles to a mixed culture containing eNSCs activated the IGF-mammalian target of rapamycin complex 1 (mTORC1) pathway in eNSCs and expanded the pool of proliferative eNSCs. These data show that the eCSF serves as a medium for the distribution of nanovesicles, including exosomes, and the coordinated transfer of evolutionary conserved molecules that regulate eNSC amplification during corticogenesis.

## Introduction

NSC amplification during development is a critical determinant of neuron density and thus brain size and function, and alterations in NSC behavior have a dramatic impact on brain function at any stage of life [1]. In light of its physiological and pathological significance, the amplification and self-renewal of NSCs is under strict regulation by extracellular signals during embryogenesis [2].

Recent studies identified the embryonic cerebrospinal fluid (eCSF) as a reservoir of signals that directly target NSCs and regulate their proliferation [3], [4]. The eCSF is essentially produced by the choroid plexus, a highly vascularized plexus of epithelial cells generated at about embryonic day 12 (e12) in rats and Carnegie stage 18 in humans [5], [6]. One of the signaling molecules identified in the eCSF is insulin-like growth factor (IGF) 2, which regulates NSC proliferation through activation of IGF-1 receptors [3], [4]. In addition, an array of intercellular and intracellular proteins has recently been identified in the human and rat eCSF [7]. The surprising wealth of intracellular molecules in the eCSF raises questions regarding their functionality for regulating the behavior of eNSCs. One intriguing possibility is that eCSF-borne signaling molecules are packaged in vesicles preventing them from being degraded and allowing signal transfer to eNSCs lining the ventricular wall.

Nanovesicles such as exosomes transfer protein, mRNA, and microRNA between cells and have emerged as a novel mechanism of intercellular communication [8], [9]. Exosomes have been found in murine eCSF [10] and in the CSF of adult humans [11]–[14]. Therefore, we tested whether nanovesicles present in the eCSF during cortical development carried elements of the IGF pathway.

Here, we identified nanovesicles, including exosomes in the eCSF of both rodents and humans that contain microRNAs and proteins, the presence of which is evolutionarily conserved between these lower and higher order mammalian species. In particular, nanovesicles contain critical components of growth and proliferation pathways, including IGF-1 receptor pathway and canonical downstream signaling mediators. Exposing mixed cultures containing eNSCs to eCSF-extracted nanovesicles led to activation of the IGF-mTORC1 signaling pathway. Thus, signaling molecules in eCSF nanovesicles offer a molecular quantum providing a multi-layer regulation of intracellular pathways as opposed to an on-or-off regulation provided by individual eCSF signaling molecules.

## Results

### Nanovesicles in the eCSF include exosomes and contain elements of the IGF pathway

**Figure 1:**
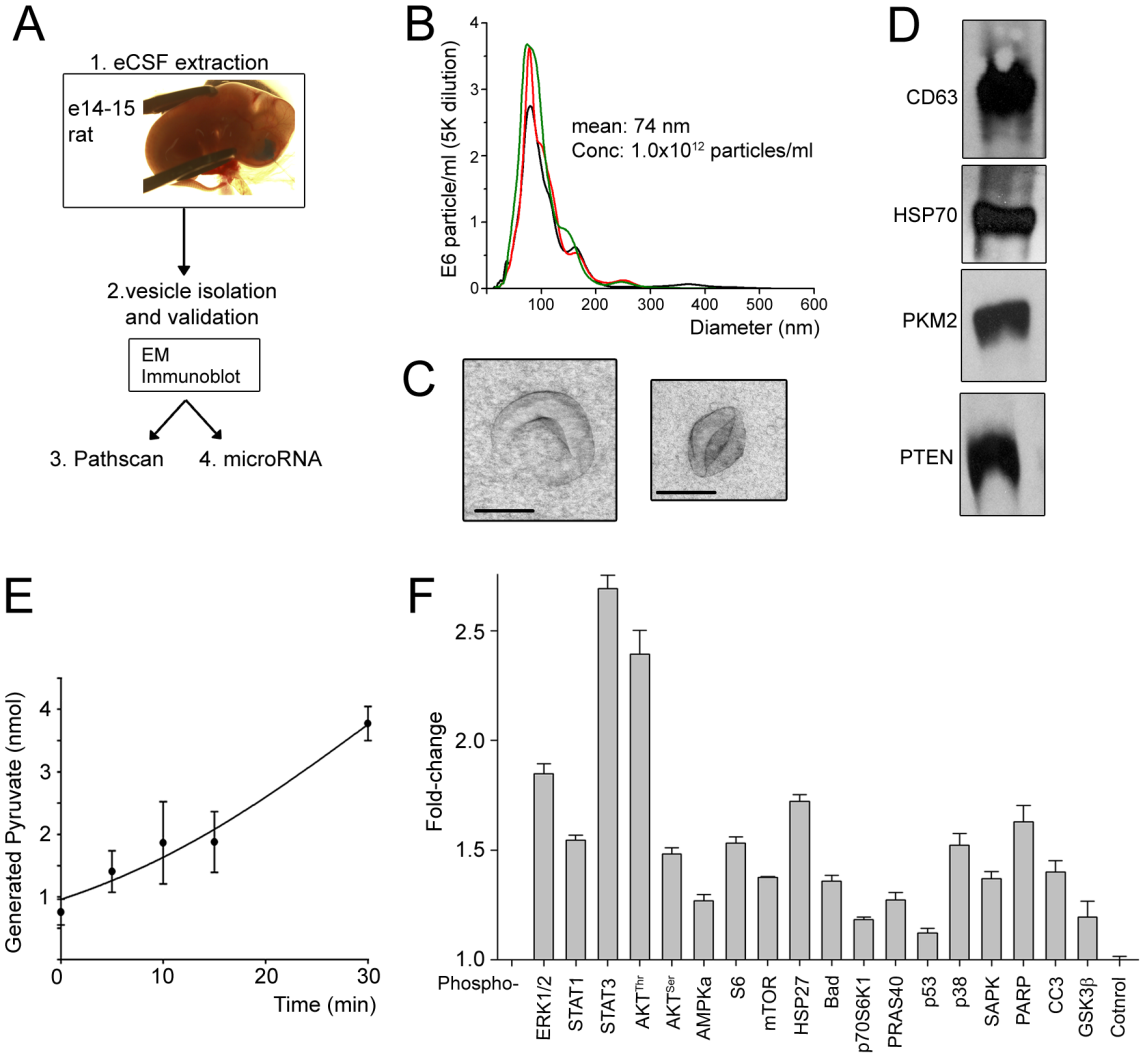
Rodent eCSF nanovesicle purification and protein expression. (**A**) Flow chart of the experimental design after CSF labeling with a tracer dye (fast green) and eCSF collection. (**B**) Histogram of size distribution of eCSF nanoparticles (per ml) determined by nanoparticle tracking analysis (NanoSight). The nanoparticles were obtained from e14 CSF from three rat litters (N = 3). The mean nanoparticle diameter was 77 nm and was obtained by Gaussian curve fitting. (**C**) Electron micrographs of rat embryonic purified nanovesicles. Scale bar: 30 nm. (**D**) Immunoblots for rat exosomal marker proteins CD63 and HSP70, and additional proteins known to be in exosomes. PTEN and PKM2. (**E**) Quantification of phosphoenol pyruvate kinase enzymatic activity determined from rat nanovesicles. (**F**) Quantification of IGF pathway-related proteins in nanovesicles isolated from e15 rat CSF using the phospho (p)-pathscan assay. Error bars: SEM. Experiments were reproduced with nanovesicles isolated from three litters (N = 3).

To determine whether vesicles are present in the CSF of e14–15 rats during the peak corticogenesis, eCSF in the lateral ventricular cavity was labeled with a tracer dye and collected with a micropipette (**Figure 1A** **and movie S1**) as previously described by others [10], [15]. CSF of e14 embryos was subjected to nanoparticle tracking (NanoSight) analysis to quantify the number and size of nanoparticles present in the eCSF. We found that the eCSF contains 1.0×10^12^ nanoparticles/ml that were 77.3±1.8 nm in diameter (N = 3) consistent with the size of exosomes and other types of nanovesicles (fractionated released midbodies, ectosomes, and cilia-released membrane vesicles) [16], [17] (**Figure 1B** and **movie S2**). To isolate nanovesicles including exosomes from eCSF, we used a polymer-based purification strategy following a previously described protocol [18], [19]. The isolated fraction was visualized by electron microscopy, which showed the presence of characteristic cup-like nanovesicles resembling exosomes ranging from 30 to 150 nm in size (**Figure 1C**) [20]. To further examine whether the isolated fraction contained exosomes, we assayed for the presence of several proteins known to be in exosomes [21]–[25]. By immunoblot we detected the presence of the exosomal marker proteins CD63 and HSP70 (**Figure 1D**). We also found PTEN, a protein consistently found in exosomes and the enzyme PKM2, previously identified in exosomes [24] (**Figure 1D**). We confirmed the absence of PTEN and PKM2 in the polymer-based solution alone and in the supernatant from the eCSF mixed with the polymer-based solution (**Figure S1A)**. In addition, we measured the activity of PKM2, a key enzyme in the glycolytic pathway. PKM2 enzymatic activity was assessed using a colorimetric assay, which quantified the amount of pyruvate generated from phosphoenolpyruvate and demonstrated a time-dependent increase in pyruvate in the exosomal fraction (**Figure 1E**). We found that the cytoplasmic protein phospholipase D1, which has not been found in exosomes [26], was absent from the isolated fraction containing nanovesicles (*e.g*, pellets, **Figure S1B**) ruling out contamination by cytoplasmic molecules. We also found no significant trace of blood contamination by measuring heme levels in the eCSF fraction (0.000107% of the heme level in blood). Collectively, these data show that nanovesicles including exosomes are present in the eCSF during corticogenesis as previously reported [10].

To explore for the presence of key components of the IGF signaling pathway in nanovesicles, we performed immunoanalysis (using the phospho (p)-pathscan assay) from e14–15 eCSF-isolated nanovesicles. We identified IGF pathway components including phospho (p)-STAT3, pAKT, pERK1/2, pSTAT1, pS6, pmTOR, and pS6K1 (all significantly increased over control at p>0.05 or more, Student's t-test, **Figure 1F**). Collectively, these results show that eCSF-isolated nanovesicles contain functionally active metabolic enzymes and key developmental regulators that are part of the IGF pathway.

### Nanovesicles from rat and human eCSF contain IGF pathway-interfering microRNAs

**Figure 2:**
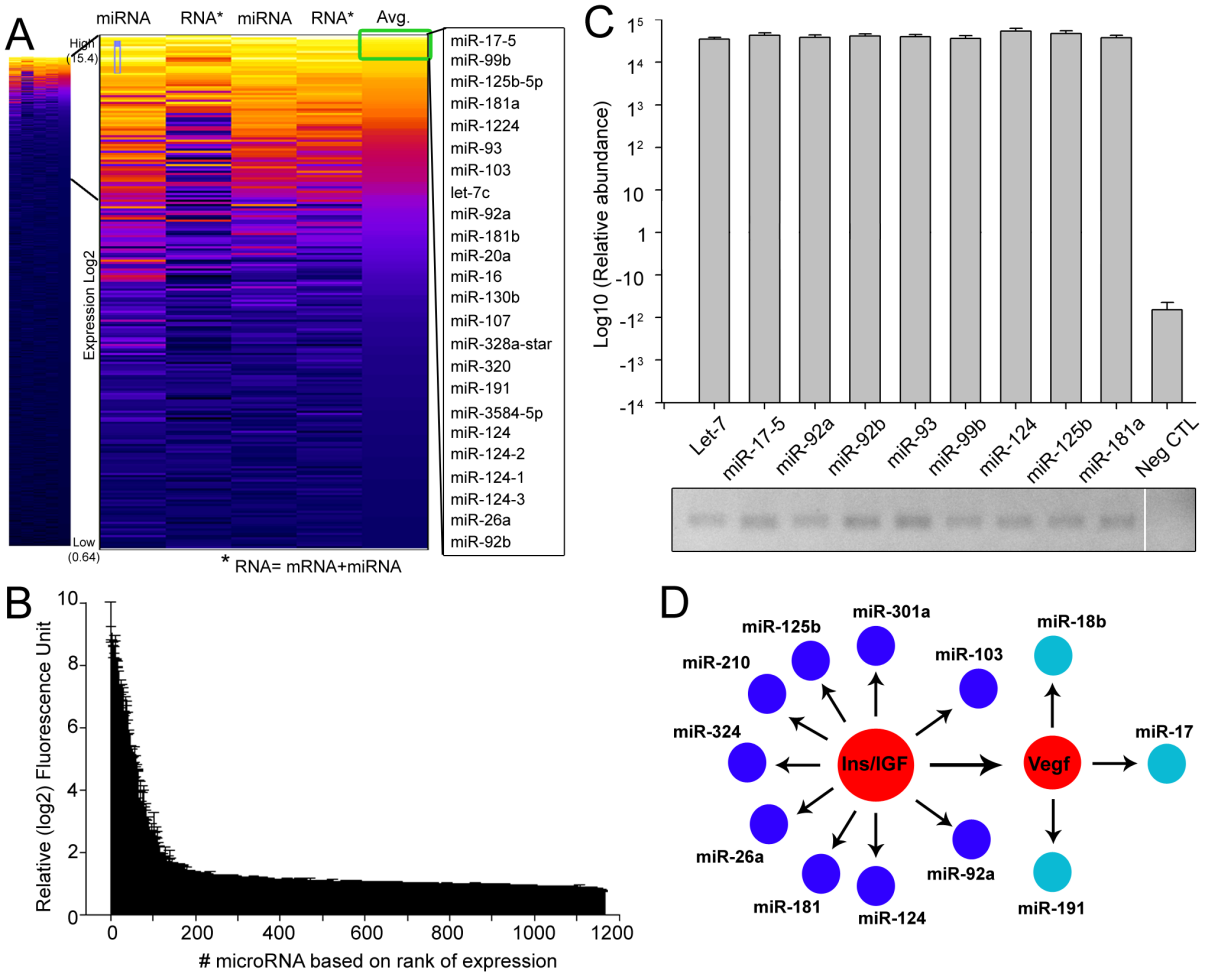
microRNA analysis of rat eCSF nanovesicles. (**A**) Heat map of microRNA microarrays from eCSF purified nanovesicles. The lighter the color (yellow) indicates higher expression whereas the darker the color (dark purple) is an indication of absence of expression. Values range from (log_2_) 0.64 (bottom) to (log_2_) 15.4 (top). The top 24 enriched microRNAs are listed on the right. (**B**) Rank expression of microRNAs based on microarray expression levels. (**C**) Quantitative (q) RT-PCR of exosomal RNA using selective exosomal microRNA primers or lacking primers (Neg CTL: negative control). Bottom: Corresponding end-point RT-PCR. (**D**) Bioinformatic analysis of microRNA interacting pathways. N = 4 litters of rats.

We next isolated RNA from eCSF nanovesicles (120.8±20.3 ng in ∼100–200 µl of CSF per rat litter). We identified the presence of several RNA species, including microRNAs and non-coding RNAs. To identify which microRNAs were present in nanovesicles, we performed microRNA microarray analysis. Out of 1166 rat probes in the array, 107 and 40 microRNAs had expression levels 4-fold and 16-fold greater than background levels, respectively (**Figure 2A and B**, and **Table S1**). The 16-fold enriched microRNAs represent 3.4% of the total probed microRNAs. In comparison a recent study identified 355 microRNAs in the developing cortex at E13 [24]. The microarray findings were further validated by confirming the presence of nine of the top microRNAs [miR-124; 125b; 17-5; 92b; 93; 92a; 181a; 99b, and Let-7] by quantitative and endpoint RT-PCR (**Figure 2C**). Bioinformatics cluster analysis of the identified 16-fold enriched microRNAs revealed that 9 microRNAs cluster within the IGF pathway (**Figure 2D**). Collectively, these data show that eCSF nanovesicles are enriched with IGF pathway-interfering microRNAs, some of which are known to play critical roles in brain development.

**Figure 3:**
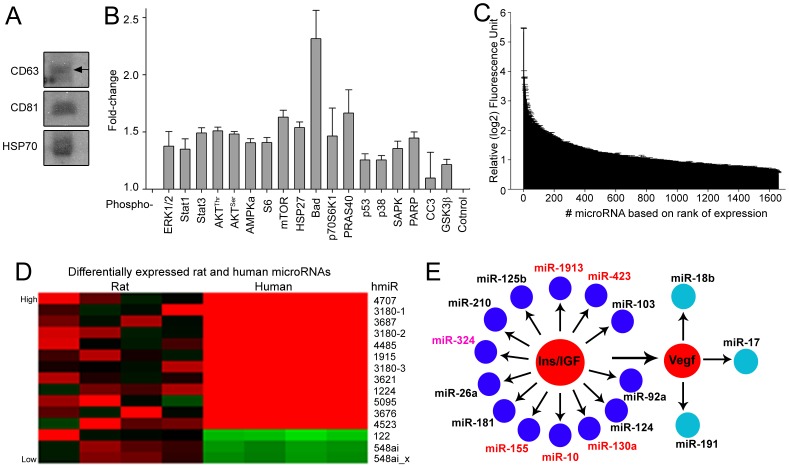
Analysis of human eCSF nanovesicles. (**A**) Western blots for CD63, CD81, and HSP70 on purified human eCSF nanovesicles. (**B**) Pathscan analysis of human eCSF nanovesicles. All except CC3 are statistically above background at p<0.05. n.s., not significant. (**C**) Rank expression of human microRNAs based on microarray expression levels. (**D**) Differentially expressed microRNAs identified by Significant Analysis of Microarray (SAM) are indicated and represented in the heat map for human nanovesicles (left) and rat nanovesicles (right). Expression values range from low (bright green) to intermediate (black) to high (red). (**E**) Bioinformatic analysis of microRNA interacting pathways identified in eCSF purified human nanovesicles. Red microRNAs: 16-fold enriched (not shared with rat microRNAs); pink microRNA: 16-fold enriched and shared with rat; black microRNAs: 4-fold-enriched in humans and 16-fold enriched in rats. N = 4 humans. Error bars: SEM.

The identification of nanovesicles in lower order mammalian species compelled us to ask whether nanovesicles are also present within the CSF of human embryos. Nanovesicles including exosomes have indeed been isolated from adult human CSF [11]–[14], but not human eCSF. CSF was isolated from post-conception week 8–12 human embryos. Isolated human eCSF was prefractionated and subjected to vesicle isolation. Western blots for CD63, CD81, and HSP70 confirmed the presence of exosomes in human eCSF (**Figure 3A**). Pathscan analysis of proteins in the eCSF nanovesicles showed the presence of components of the IGF pathway, albeit at different relative levels from those in rats (**Figure 3B**). From human eCSF nanovesicles, we prepared microRNA fractions and subjected samples to array analysis. We identified 167 microRNAs that were expressed 16-fold greater than background (**Figure 3C**, and **Table S2**).

Microarrays contain probes for humans and rats, which allows for cross-species comparison of nanovesicle microRNAs. We found that the most highly enriched human microRNAs were also present in rats, including miRNA-124, 125b, and 320a/b. In addition, only 15 microRNAs were significantly differentially expressed between rats and humans (**Figure 3D**). 73.3% of differentially expressed microRNAs had no comparable rat homologues (11/15). We next performed bioinformatic analysis of the top 167 microRNAs (16-fold enriched) found in human eCSF nanovesicles to identify pathway clusters that may be integral to fetal human development. As in rodents, this analysis identified the presence of an IGF/Insulin-activated microRNA node comprised of miR-1913, 423, 155, 130a, and 10 (red on diagram) as well as miR-324 (pink), which was shared with the rat IGF/Insulin node (**Figure 3E**). In addition, all rat IGF/Insulin node microRNAs (except miR-301a) were present in humans (black in **Figure 3E**), albeit at a lower level (4-fold instead of 16-fold enriched).

### eCSF nanovesicles applied to a mixed eNSC-neuroblast culture activate the IGF-mTORC1 signaling pathway in NSCs *in vitro*

**Figure 4:**
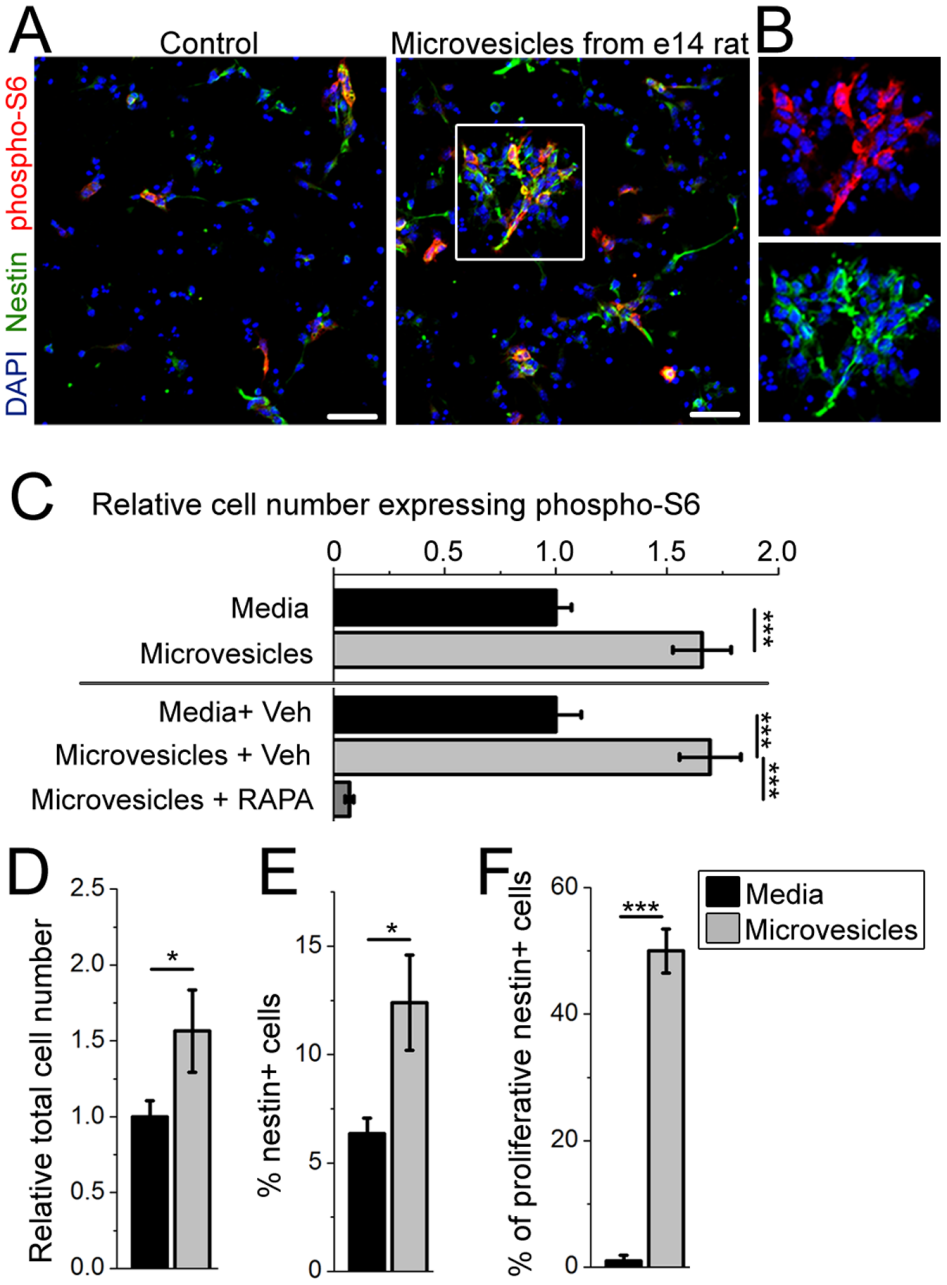
Nanovesicles from eCSF increase IGF-mTORC1 activity in eNSCs *in vitro*. (**A**) Control and nanovesicle-treated eNSCs *in vitro* immunostained for phospho-(p) S6 (red), and nestin (green), and counterstained for the nuclear marker DAPI (blue). (**B**) Zoom of the image in the white square in (A). (**C**) Number of phospho-S6-positive cells relative to total with or without nanovesicle application (N = 3 and 4 cultures, 2–4 litters for nanovesicle extraction). Experiments were reproduced in the presence of vehicle (DMSO) or 100 nM rapamycin (N = 3 each). (**D**) Relative total cell number (N = 3 each). (**E**) Percentage of nestin-positive eNSCs (N = 3 each). (**F**) Percentage of Ki67- and nestin-positive eNSCs (N = 9 control and 3 with nanovesicle). *: p<0.05, **: p<0.01, ***: p<0.001 with Student's t test or one way ANOVA. Scale bars: 200 µm (A and B). Error bars: SEM.

Considering that eNSCs are in direct contact with the eCSF, we explored whether eCSF nanovesicles are capable of activation the IGF pathway in eNSCs. Cortical NSCs from e12.5 mice were cultured in a defined media, which is free of eCSF nanovesicles, for 24 hours as previously reported [27]. The culture contained a mixed cell population mostly composed of eNSCs and neuroblasts (doublecortin-positive) and eventually neurons (NeuN-positive) generated over time (data not shown). Mouse e14 CSF purified nanovesicles or FBS-free media subjected to nanovesicle isolation (control) were used to treat eNSCs for 24 hours. Data were obtained from 3 and 4 cultures and 2-4 litters for nanovesicle extraction as specified in the figure legend. We then examined the phosphorylation of the ribosomal protein S6 (serine S240/244), which is an established readout of IGF-mTORC1 pathway activity [28]. Treatment of eNSCs with eCSF nanovesicles resulted in a significant 66% increase in the number of phospho (p) S6-positive cells (p<0.001, Student's t-test, **Figure 4A-C**). The increase in pS6 was almost completely ablated by rapamycin, a mTORC1 inhibitor (**Figure 4C**). eNSC exposure to eCSF nanovesicles also resulted in a significant 57% increase in the total number of cells (p<0.05, **Figure 4D** **)**. However, rapamycin did not alter the nanovesicle-induced increase in total cell number, suggesting that activation of an mTORC1-independent pathway is responsible for cell number regulation (data not shown). The change in total cell number was not due to an increase in the percentage of neurons examined by NeuN immunostaining (data not shown). Rather, there was a 2-fold increase in the percentage of eNSCs in culture (from 6% to 12%, p<0.05, **Figure 4E**) and a significant increase in the percentage of proliferative (Ki67-positive) nestin-positive cells (*i.e*., eNSCs) from 1% to 50% (p<0.001, **Figure 4F**). As an additional control, we subjected cells to nanovesicle-depleted eCSF resulting in only a 15% increase in the number of proliferative eNSCs (N = 3 cultures, data not shown). To further assess specificity of the effect of eCSF nanovesicles on eNSCs, we also treated the mixed eNSC cultures with nanovesicles isolated from cortical e17 neurons cultured for 2 days. Nanovesicles isolated from this neuronal culture had no effect on the number of proliferative eNSC, but affected neuronal differentiation (N = 3 cultures, data not shown). These results demonstrate that eCSF nanovesicles applied to a mixed eNSC-neuroblast culture activate the PI3K-mTORC1 signaling pathway downstream of IGF signaling in eNSCs that was accompanied by alteration in their proliferative behavior independent of mTORC1.

## Discussion

Here, we report that eCSF nanovesicles contain microRNAs and proteins, the presence of which is evolutionarily conserved between lower order (rodent) and higher order (human) mammalian species. Intriguingly, the set of proteins and microRNAs contain critical and functional components of the IGF signaling pathway that regulate mTORC1 activity in eNSCs. Finally, exposing eNSCs to eCSF nanovesicles led to NSC amplification independent of mTORC1. Thus, our work implicates nanovesicles in the eCSF as critical determinants of eNSC development through the transfer of an evolutionary conserved molecular community providing multiple layers of IGF pathway regulation.

The eCSF has recently been reported to contain an unanticipated large repertoire of molecules, including cytoplasmic and nuclear proteins [7]. A proteomics-based study further emphasizes the importance of the eCSF during embryogenesis and highlights the necessity to have carriers for eCSF intracellular proteins to be protected from degradation and remain functional [4]. In fact, an earlier study in e10.5–e12.5 mice reported the presence of particles of different sizes (∼600 nm and 50–80 nm) including exosomes in the ventricular fluid that were presumably released by neuroepithelial cells based on the expression of CD133 (prominin 1) in these nanovesicles [10]. Our study further supports the presence of nanovesicles, including exosomes in the eCSF that are present in large quantities (trillions/ml). There are no studies using nanoparticle tracking to estimate the quantity of nanovesicles in embryonic or adult CSF. Nevertheless, the number of vesicles calculated in blood from patients is 1.07×10^10^ and increases by four-fold in ovarian cancer patients [29]. In normal rodent eCSF, we have thus nearly 100 times more nanoparticles than in the blood of adult humans. We identified nanovesicles as exosomes based on their size (77 nm diameter) and marker expression such as CD63. In addition, the isolated eCSF nanovesicles contained molecules such as PTEN and PKM2 found in exosomes as listed in the online database ExoCarta.org. The greatest risk of contamination is from the blood since a sharp glass pipette is inserted through the cortical tissue to reach the lateral ventricle. However, we used a method described by others that showed no visible contamination by cells, including red blood cells [7], [15]. In addition, we found that there was no significant trace of heme in the collected eCSF.

The nanovesicles isolated from eCSF from both rodents and humans contained a repertoire of proteins and non-coding RNA of different sizes that include microRNAs. We identified an overrepresentation of proteins and microRNAs that interfere with the IGF signaling pathway. This finding is consistent with the critical function of the IGF signaling pathway on eNSC development and corticogenesis [3], [30]. Interestingly, the eCSF nanovesicles contained molecules that can exert a tight regulation of the IGF pathway activity by either increasing (*e.g*., phospho-AKT) or decreasing its activity (*e.g*., PTEN). Changes in the relative level of individual molecules inside the nanovesicles could influence the outcome of this tug of war exerted on the IGF pathway. Thus, the molecules in eCSF nanovesicles offer a multi-layer regulation of intracellular pathways and genetic programs as opposed to an on-or-off (binary) regulation provided by individual eCSF signaling molecule.

A surprising result of our study is that the majority of microRNAs, which were identified in human eCSF nanovesicles were also detected in rat eCSF nanovesicles. Nevertheless, a small cohort of microRNAs showed differential expression beyond what was predicted by significance analysis of microarray (SAM). The differential expression of these microRNAs could potentially alter the timing, rates, or types of divisions in eNSCs. For example, miR-1915 has been reported to be expressed in human eNSCs where it regulates a pro-neurogenic transcript, Notch-1 [31]. Ultimately, a combination of targets of each microRNA in their respective species could account for the fine tuning of eNSCs during cortical development.

Exposing e12.5 NSCs to eCSF nanovesicles led to the activation of the PI3K-mTORC1 signaling pathway downstream IGF signaling and impacted the development of eNSCs. In particular, nanovesicles significantly increased the number of total as well as proliferative eNSCs *in vitro*. The effect of nanovesicles on eNSC proliferation was independent of mTORC1 activity since it persisted in the presence of rapamycin. This is consistent with a recent study reporting that mTORC1 activation does not induce neonatal NSC to proliferate [32]. The lack of rapamycin effect does not rule out a contribution of nanovesicle IGF signaling on eNSC proliferation. Indeed, IGF signaling can activate several pathways including ERK and AKT signaling that could contribute to the proliferation effect of nanovesicles on eNSC amplification independent of mTORC1 [33]. Considering that the e12.5 culture also contained neuroblasts, it is possible that the effect of nanovesicles on eNSCs was indirect via the release of unknown factors form e12.5 neuroblasts. Nonetheless, these data suggest that molecules in the nanovesicles are functional and interfere with the IGF signaling pathway in embryonic neural cells, possibly directly in eNSCs.

Our data open a new series of questions that are outside the scope of this first study. For example, it is conceivable and likely that the composition of nanovesicles changes over time and thus differently affects the activity of the IGF signaling pathway and eNSC development. future work is also require to identify the sources of eCSF nanovesicles. The choroid plexus is a likely contributor to nanovesicle synthesis and release [13], but other sources include the eNSC themselves, neuroblasts, and perhaps endothelial cells. Developing novel methods to remove endogenous exosomes from the eCSF or change the levels of selective molecules in endogenous exosomes are additional directions to be pursued.

Collectively, our findings set the stage for future studies aimed at examining the role of eCSF nanovesicles and more specifically exosomes on eNSC development as well as the influence of individual molecules specifically on the IGF signaling pathway in eNSCs. At the present stage, our data are underscored by the fact that alterations in the IGF1-PI3K-AKT pathways are observed in tumors of neural origin and disorders associated with severe neurological symptoms such as Tuberous sclerosis complex (TSC) and hemimegalencephaly [34]–[36]. Defects in the molecular content of nanovesicles could contribute to non-cell autonomous mechanisms of disease progression in neurodevelopmental disorders. Therefore, although exosomes have been identified as candidate biomarkers of schizophrenia and epilepsy, the potential contribution of nanovesicles and more specifically exosomes to the origin or amplification of neuropathological development should be considered.

## Materials and Methods

### Ethics statement

The human eCSF was obtained from the following Research tissue Bank: the MRC-Wellcome Trust Human Developmental Biology Resource (HDBR) at URL: http://www.hdbr.org/. The eCSF was obtained with approval by the institutional review boards at the Yale University School of Medicine and University of Newcastle, from which tissue specimens were obtained. Tissue was handled in accordance with ethical guidelines and regulations for the research use of human brain tissue set forth by the NIH and the University of Newcastle. Donors are asked to give explicit written consent for the fetal material to be collected. Animal protocols were approved by the Yale University Institutional Animal Care and Use Committee.

### Animal use

Experiments were performed on timed pregnant CD1 mice or Sprague Dawley rats from Charles River. Mice and rats were of either gender. All experiments were reviewed and approved by the Yale University Institution for Animal Care and Use committee.

### Embryonic CSF isolation, tracking analysis, and vesicle isolation

Pregnant dams (embryonic day 14–18) were injected with Buprenorphine 30 minutes prior to anesthesia by isoflurane. Animals were shaved, laparotomy performed, and embryos individually extracted by making an incision through the uterine horn. Embryos were transferred on ice and removed from placental sac. The tracer dye Fast green was dissolved in saline solution (0.9% w/v, Sigma-Aldrich) and was injected into lateral ventricles using a pulled glass pipette. eCSF was then aspirated using manual pressure immediately following injection of fast green. eCSF was sequentially pooled on ice in a pre-chilled 1.5 ml reaction tube and immediately centrifuged for 15 minutes at 3,000 g to remove cellular debris and supernatant transferred to a new tube. The Nanosight LM10 nanoparticle characterization system (NanoSight, NanoSight Ltd, UK) equipped with a blue laser (638 nm) illumination was used for real-time characterization of the number and size of the particles. Mean size and particle concentration values were calculated by the nanoparticle tracking software. which allows analysis of video images of the particle movement under Brownian motion captured by Nanosight LM10 and calculation of the diffusion coefficient, sphere equivalent, and hydrodynamic radius of particles by using the Strokes-Einstein equation. Alternatively, isolated eCSF was centrifuged at 3,000 g and supernatant was then mixed with equal volumes ExoQuick Exosome Precipitation (Systems Biosciences, Mountain View, CA) solution and placed on a rotisserie overnight at 4°C. Samples were subsequently centrifuged at 3,000 g at 4°C for 30 minutes. Supernatant was removed and cell pellets were used for protein or RNA analysis or incubated with Neurobasal A to treat cells.

### Heme measurements

Heme levels were assessed by a colorimetric assay according to the manufacturer's instructions (Cayman Chemical, Ann Arbor, MI). A standard curve was generated by adding hemoglobin standards to hemoglobin detector from 0.016 g/dl to 0.400 g/dl. eCSF aspirates were also added to hemoglobin detector. Solution was added to 96 well plates, covered, and incubated for 15 minutes at room temperature. Absorbance was measured at 580 nm using a standard plate reader. Assuming that hemoglobin levels for rats is 14 g/dl, samples have a mean of 0.000107% heme compared to that in the blood.

### Electron microscopy

Electron microscopy was performed by the Yale imaging core facility. Briefly, purified vesicles were resuspended in 4% wt/vol paraformaldehyde in phosphate buffered solution (pH 7.4) and embedded for 20 minutes at room temperature in a formvar-carbon-coated grid. The embedded vesicles were washed in phosphate buffered saline (PBS), fixed in 1% gluteraldehyde for 5 minutes, and stained with saturated aqueous uranyl oxalate. Samples were subsequently embedded in 0.4% wt/vol uranyl acetate and 1.8% wt/vol methylcellulose on ice for 10 minutes. Samples were dried at room temperature prior to visualization with a Carl Zeiss 910 electron microscope (Carl Zeiss Microscopy, Thornwood, NY).

### Exosomal RNA and microRNA isolation

To isolate total RNA, Trizol was added to vesicle pellets and vortexed for 15 seconds. Following a 5 minute-incubation at room temperature, chloroform was added to each tube. Samples were subjected to vigorous manual shaking for 15 seconds, incubated for an additional 5 minutes at room temperature, and centrifuged for 15 minutes at 12,000 g at 4°C. The upper aqueous phase was transferred to a fresh tube and isopropyl alcohol added to precipitate RNA. Samples were incubated for 30 minutes at 4°C then centrifuged for 10 minutes at 12,000 g at 4°C. Supernatant was discarded and pellets were washed with 70% ethanol, vortexed, and centrifuged for 5 minutes at 12,000 g at 4°C. The previous step was repeated and samples dryed and rehydrated in RNase/DNase free DEPC treated water. For microRNA isolation, vesicle pellets were incubated with Lysis buffer for 5 minutes at room temperature following 15 seconds of vortexing. 100% ethanol was added to each sample and vortexed for 10 seconds. Samples were then transferred to a spin column (Systems Biosciences, Mountain View, CA) and centrifuged for 15 minutes at 12,000 g at 4°C. Flow through was discarded and wash buffer was added. Samples were centrifuged at 12,000 g for 1 minute and samples were washed two more times. Spin columns were placed into fresh prechilled 1.5 ml elution tubes. Elution buffer was added directly to membranes of spin columns and centrifuged at 2,000 g for 2 minutes. Eluate was added back to spin columns and a final 1 minute centrifugation step was performed at 12,000 g at 4°C.

### ImmunoBlot and Pathscan

Vesicles were lysed in 100 µl Ripa Buffer and equal amounts 2x Laemmli buffer. In the case of human fetal samples, protein was purified following Trizol extraction using a chloroform-methanol precipitation and processed as above. Equal amounts of vesicles (in terms of number of particles) were resolved by standard electrophoresis conditions on 7.5% polyacrylamide precast mini-Protean TGX gels and transferred to polyvinylidene difluoride membranes. Membranes were rinsed in TBST (Tris-buffered saline, 0.1% Tween 20) for 5 minutes at room temperature and subsequently blocked in 5% w/v nonfat milk in TBST for 18 hours overnight at 4°C. Following three rinses (each of 5 minutes) at room temperature in TBST, samples were incubated for 1 hour at room temperature or overnight at 4°C with the following antibodies: PKM2 (Cell Signaling Technology, Danvers, MA, 1∶000), HSP70 (Systems Biosciences, Mountain View, CA, 1∶500), CD63 (Systems Biosciences, Mountain View, CA, 1∶500), PTEN (Cell Signaling Technology, Danvers, MA, 1∶1000), and phospholipase D1 (Cell Signaling Technology, Danvers, MA, 1∶1000). Following an additional three rinses each of 5 minutes in TBST, samples were incubated for 1 hour at room temperature with donkey or goat anti-rabbit antibodies in blocking buffer and then subjected to four 15-minute washes in TBST and visualized by enhanced chemiluminescence. In the case of the PathScan® Intracellular Signaling Array Kits (Fluorescent Readout, Cell Signaling Technology, Danvers, MA), 1.50 ml RIPA buffer was used to lyse vesicles. Assembled arrays were incubated in blocking buffer for 1 hour at room temperature on an orbital shaker. Array blocking buffer was replaced with 80 µl of vesicle extract and incubated overnight at 4°C. Lysate was removed and replaced with array wash buffer four times at room temperature for 5 minutes each time. Arrays were incubated with DyLight 680-linked to streptavidin for 30 minutes at room temperature on an orbital shaker, washed for 5 minutes at room temperature four times with wash buffer and imaged using a LiCor Odyssey imaging system (Li-Cor Biosciences, Lincoln Nebraska).

### Enzymatic assay

A colorimetric pyruvate kinase assay was performed by measuring pyruvate generation in comparison to pyruvate standards according to manufacturer's recommendations (BioVision Inc., Milpitas, California). Briefly, samples, standards, or controls were added to Assay buffer. Standard curves were generated in triplicate with 0.2–10 ng of pyruvate. Diluted samples were then added to substrate mix, enzyme mix, and OxiRed Probe. Measurements were performed on 96 well plates containing samples by determining the optical density (OD) at 565 nm with a microplate reader (Model 550; Bio-Rad, Carlsbad, CA).

### MicroRNA microarrays

Pooled samples from 1–2 litters of purified exosomal RNA or microRNA were processed by the Yale Center for Genome Analysis. Approximately 500 ng microRNA was subjected to labeling and ligation with the FlashTag Biotin Labeling kit (Affymetrix, Santa Clara, CA) and subjected to ELOSA QC analysis for ensured labeling. Samples were hybridized to Affymetrix Genechip miRNA 3.0 arrays (Affymetrix, Santa Clara, CA) for 16 hours at 45°C, washed, stained with an affymetrix fluidics station, and scanned using a 16-bit Genechip scanner 3000 (Solid State 532 nm Diode-pumped Frequency Doubled neodymium-doped yttrium aluminum garnet green laser). Samples were analyzed using the Affymetrix genechip Command Console software.

### Bioinformatic pathway and cross species analysis

MicroRNAs with a greater than 16-fold expression were subjected to Ingenuity Systems IPA analysis. Pathways with statistically significant representations were included. Microarray software suite MultiExperiment Viewer v4 software, which is part of the TM4 software suite available freely from the J. Craig Venter Institute (http://www.tm4.org/index.html website) was used for visualizing and calculating significance analysis of microRNA microarrays and cross species analysis, and in particular generating Significance Analysis of Microarrays (SAM) graphs.

### qRT-PCR

Approximately 250 ng nanovesicle-purified microRNA was combined with polyA buffer, 25 mM MnCl_2_, ATP, and polyA polymerase along with SeraMir 3′ adaptor oligo (Systems Biosciences, Mountain View, CA) and incubated at 60°C for 5 minutes. Samples were then incubated for 2 minutes at room temperature and placed on ice. Next, RT master mix, water, a 5′ Seramir switch oligo, and reverse transcriptase were combined with samples and incubated at 42°C for 30 minutes and at 95°C for 10 minutes to yield cmiDNA. Approximately 25 ng cmiDNA was combined with IQ™ SYBR green supermix, sterile DNase/RNase free water, a universal 3′ Linker targeted primer, and a sequence specific cmiDNA primer (BioRad Laboratories, USA). Samples were subjected to 45 rounds of amplification on a BioRad MyCycler with an initial hot start reaction. MicroRNAs were detected within 20 cycles and appropriate template amplification was verified by melt curve analysis along with running out final products on 2% agarose gels stained with ethidium bromide.

### Embryonic NSC and neuronal culture and immunocytochemistry

Embryonic day 12.5 mouse pups were removed from anesthetized mice following laparotomy and placed into 4°C Neurobasal A media on petri dishes chilled on wet ice. Dissections were performed as previously described and demonstrated [27]. Skin and mesenchymal layers were removed and telencephalic nanovesicles placed into 0.05% trypsin with 0.02% EDTA and 0.2% BSA in HBSS for 20 minutes in a 37°C incubator. Equal volumes of 1 mg/ml soybean trypsin inhibitor in HBSS were added followed by trituration with three pasture pipettes fire-polished with sequentially decreased bore size to dissociate clumps. Cells were placed on laminin-coated coverslips in 12 well plates in complete media (Neurobasal A, Glutamax, and Penicillin/Streptomycin, Life Technologies, Grand Island, NY). The following day, complete media was replaced and when indicated, supplemented with 333×10^6^ nanovesicles/well. We used two groups of control experiments. For one group of controls, cells were cultured with FBS-free media subjected to the same vesicle isolation steps as for the eCSF. For the second group of controls, cells were subjected to vesicle-depleted eCSF (*i.e.*, the supernatant).

Twenty four hours later cells were fixed by adding equal volumes of 37°C fixative solution (4% paraformaldehyde in 300 mM sucrose, phosphate buffered saline) directly to the wells. Half of the solution was then removed from each well and replaced for a total of four times and incubated at 37°C for 15 minutes. Coverslips were then washed three times for 5 minutes at room temperature in wash buffer (PBS, 0.1% Tween-20). Coverslips were blocked in antibody buffer (PBS, 2% BSA, 0.1% Tween-20) with 0.1% Triton-X 100 and then washed again three times in wash buffer for 5 minutes at room temperature. Coverslips were then incubated in antibody buffer with antibodies against: phospho-S6 240/244 (Cell Signaling Technology, Danvers, MA, 1∶500), Nestin (Novus Partners Inc. NY, 1∶500), Ki67 (Vector Labs, VP-RM04, 1∶500), and NeuN (Millipore, MAB377, 1∶500). Coverslips were washed three times for 5 minutes at room temperature in wash buffer and then incubated in antibody buffer with the appropriate secondary antibodies (1∶1000, Life Technologies, Grand Island, NY). Following four 15 minute-washes at room temperature in wash buffer, coverslips were mounted in ProLong^R^ Gold antifade with DAPI (Life Technologies, Grand Island, NY) on superfrost microscope slides (Thermo Scientific, Logan, UT).

Pure cortical e17 neuronal culture were obtained as previously described [37]. Cells were cultured for two days prior to collecting the supernatant for vesicle isolation as described above.

### Statistical analysis

Statistical analyses for bioinformatics and microarray experiments were performed using MASCOT, Ingenuity, or TM4: Microarray Software. Student's t-test or one-way ANOVA was performed for *in vitro* experiments.

## Supporting Information

Figure S1 (A) Immunoblots for PKM2 and PTEN in rat pellets (containing eCSF nanovesicles) and in the supernatant from the eCSF mixed with the Exoquick polymer-based solution, and in the polymer-based solution alone. This latter data suggest that there is no cross reactivity between PTEN and PKM2 antibodies and the extraction polymer. **(B)** Immunoblots for the non-exosomal protein phospholipase D1 in whole cell lysate of Neuro2a cells and in eCSF pellets containing the nanovesicle fraction.
(TIF)

Table S1 Rat microarray data (excel sheet).
(XLSX)

Table S2 Human microarray data (excel sheet).
(XLSX)

Movie S1 Movie illustrating the isolation of eCSF from e14 embryo still attached to the mother.
(AVI)

Movie S2 Live monitoring of nanoparticles in suspension at 21°C in the NanoSight sample chamber. Total frames: 1195 with acquired at 19.93 frames per sec. 187 nm/pixel.
(AVI)
