# Supplementary figures and images for: Embryonic Cerebrospinal Fluid Nanovesicles Carry Evolutionarily Conserved Molecules and Promote Neural Stem Cell Amplification
Source: PLoS One. 2014 Feb 12;9(2):e88810. doi: 10.1371/journal.pone.0088810 (PMC3923048; doi:10.1371/journal.pone.0088810)

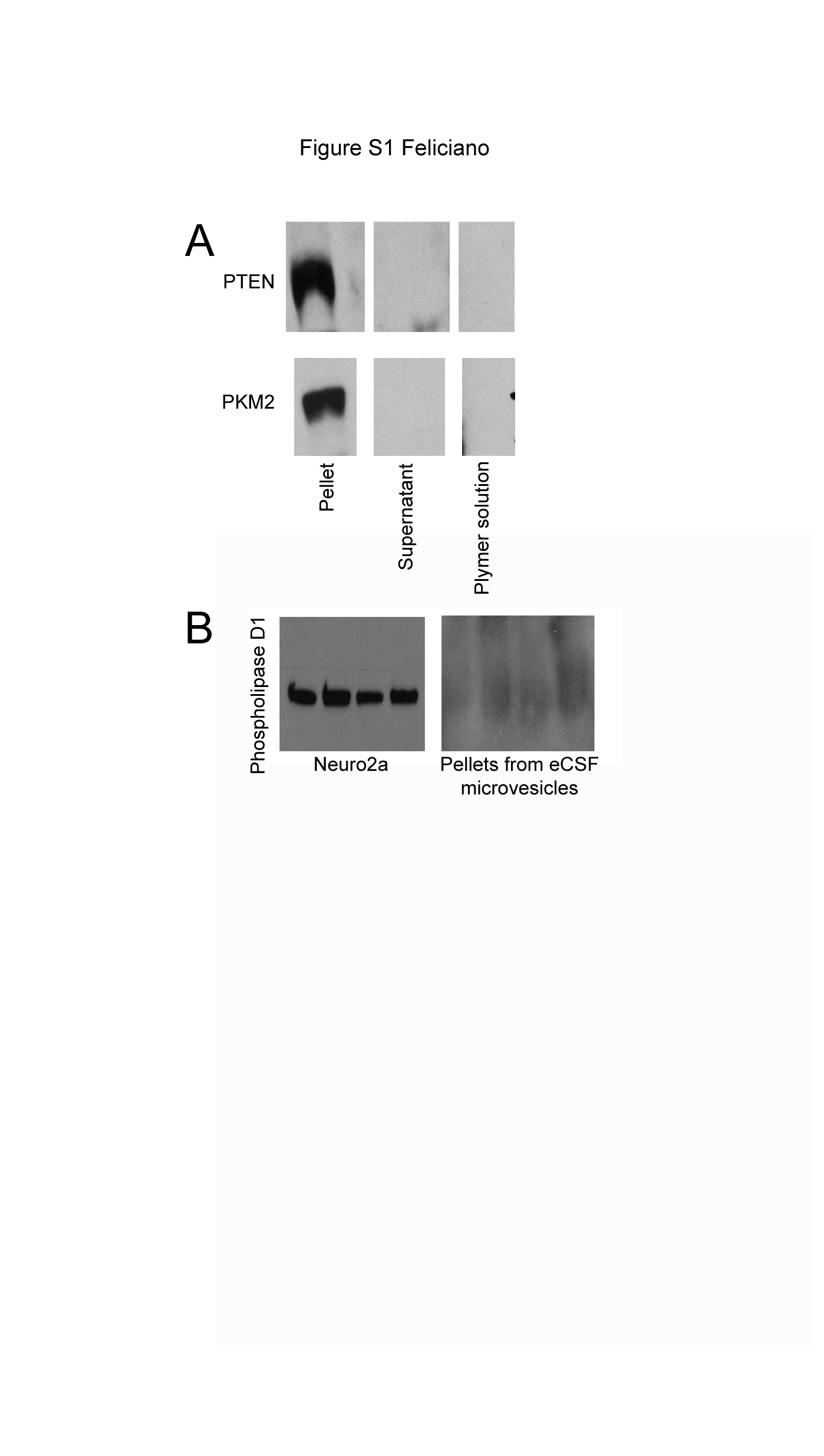

Supplement: Figure S1 — (A) Immunoblots for PKM2 and PTEN in rat pellets (containing eCSF nanovesicles) and in the supernatant from the eCSF mixed with the Exoquick polymer-based solution, and in the polymer-based solution alone. This latter data suggest that there is no cross reactivity between PTEN and PKM2 antibodies and the extraction polymer. (B) Immunoblots for the non-exosomal protein phospholipase D1 in whole cell lysate of Neuro2a cells and in eCSF pellets containing the nanovesicle fraction. (TIF) [file pone.0088810.s001.tif]
